# Linking α-synuclein-induced synaptopathy and neural network dysfunction in early Parkinson’s disease

**DOI:** 10.1093/braincomms/fcac165

**Published:** 2022-06-22

**Authors:** Aishwarya S Kulkarni, Matthew R Burns, Patrik Brundin, Daniel W Wesson

**Affiliations:** Department of Pharmacology & Therapeutics, University of Florida, 1200 Newell Dr, Gainesville, FL 32610, USA; Department of Neurology, University of Florida, 1200 Newell Dr, Gainesville, FL 32610, USA; Norman Fixel Institute for Neurological Disorders, University of Florida, 1200 Newell Dr, Gainesville, FL 32610, USA; Pharma Research and Early Development (pRED), F. Hoffman-La Roche, Little Falls, NJ, USA; Department of Pharmacology & Therapeutics, University of Florida, 1200 Newell Dr, Gainesville, FL 32610, USA; Norman Fixel Institute for Neurological Disorders, University of Florida, 1200 Newell Dr, Gainesville, FL 32610, USA

**Keywords:** neuron, synapse, oscillation, neural network, prodrome

## Abstract

The prodromal phase of Parkinson’s disease is characterized by aggregation of the misfolded pathogenic protein α-synuclein in select neural centres, co-occurring with non-motor symptoms including sensory and cognitive loss, and emotional disturbances. It is unclear whether neuronal loss is significant during the prodrome. Underlying these symptoms are synaptic impairments and aberrant neural network activity. However, the relationships between synaptic defects and network-level perturbations are not established. In experimental models, pathological α-synuclein not only impacts neurotransmission at the synaptic level, but also leads to changes in brain network-level oscillatory dynamics—both of which likely contribute to non-motor deficits observed in Parkinson’s disease. Here we draw upon research from both human subjects and experimental models to propose a ‘synapse to network prodrome cascade’ wherein before overt cell death, pathological α-synuclein induces synaptic loss and contributes to aberrant network activity, which then gives rise to prodromal symptomology. As the disease progresses, abnormal patterns of neural activity ultimately lead to neuronal loss and clinical progression of disease. Finally, we outline goals and research needed to unravel the basis of functional impairments in Parkinson’s disease and other α-synucleinopathies.

## Teaser

A common conceptualization of Parkinson’s disease is that α-syn aggregation leads to cell death, followed by symptomology. Here, Kulkarni *et al*. propose a novel ‘synapse to network prodrome cascade’ wherein in the absence of overt cell death, pathological α-syn induces synaptic loss and aberrant network activity, underlying prodromal non-motor symptoms.

## Graphical Abstract

**Graphical Abstract:**
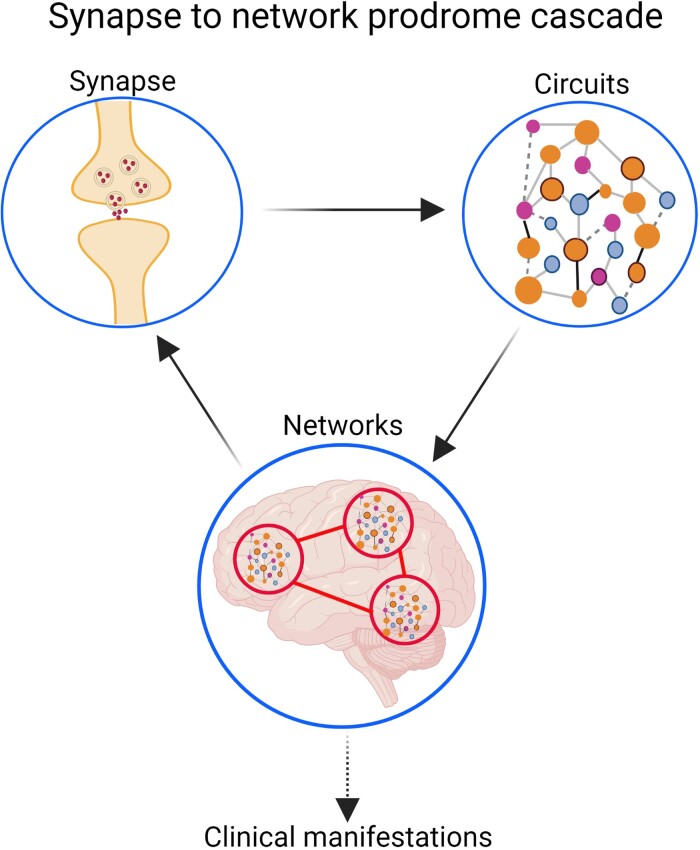

## A starting framework to understand dysfunction in Parkinson’s disease

Parkinson’s disease is the second most common neurodegenerative disease, affecting nearly 1 million people in the USA and more than 6 million people worldwide.^1^ Death of substantia nigra (SN) neurons and intracellular inclusions composed of α-synuclein (α-syn) are the neuropathological hallmarks of Parkinson’s disease.^2,3^ This pathology is thought to lead to the classic motor symptoms of rest tremor, bradykinesia, rigidity and balance impairments, which make up the core diagnostic criteria of Parkinson’s disease. While motor abnormalities mark the formal onset of Parkinson’s disease, non-motor features, including olfactory perceptual deficits, sleep disturbances, cognitive impairments and autonomic dysfunction are routinely observed in the prodromal stages of the disease.^4^

A key driver of all complex neurologic functions including cognition, sleep and motor control is the communication of neurons between spatially distributed networks, in the form of neural oscillations.^5–12^ This oscillatory activity is generated in part by synaptic transmission between groups of neurons,^13^ and changes in synaptic dynamics can lead to alterations in neuronal firing and thus aberrant neural network activity.^14–17^

The onset of Parkinson’s disease pathology is considered to start at least 20 years before detectable motor abnormalities.^18,19^ Braak *et al*. proposed a staging scheme wherein Parkinson’s disease-like pathological α-Syn aggregation and dysfunction begins in Stages 1–2 in the dorsal motor nucleus of the vagus nerve and the olfactory bulb (OB), eventually spreading to the locus coeruleus and raphe nucleus. This is accompanied by olfactory deficits, dysautonomia, rapid eye movement behaviour disorder and mood disorders such as depression, apathy and anxiety. Pathology then spreads to the SN and nucleus of Meynert in early Stage 3, initially without clinically appreciable motor impairments. Stages 1–3 are commonly referred to as the pre-symptomatic or prodromal phase. As discussed in more detail later, this prodromal phase is marked by cellular and network dysfunction underlying preclinical symptomology without overt cell death. Eventually the pathology spreads throughout the midbrain and into the limbic areas in Stage 4, whereupon it is believed to result in classical motor symptoms including tremors, stiffness and slowness, and in late Parkinson’s disease, to neocortical regions leading to progressive cognitive and affective impairments. Stages 4 to 6 are commonly referred to as the symptomatic phase and, marked primarily by massive cell death, as well as cellular and network dysfunction.

In this review, we summarize insights into the cellular (synaptic) and network-level dysfunction observed in prodromal Parkinson’s disease to develop a framework for understanding the mechanisms underlying symptoms present during this prodromal stage. We propose two broad, not mutually exclusive, causal factors underlying network dysfunction in Parkinson’s disease. First, cell-autonomous mechanisms of individual neurons which when collectively operating within a neural network, result in abnormalities at the neural network level—like singers in a chorus singing out of tune. Second, cell death may remove components of the network resulting in aberrant neural network activity^20^—like removing several members of a chorus resulting in disharmony. Here we discuss key lines of evidence supporting these two drivers of altered network activity and synthesize a novel framework where such changes ultimately lead to impairment in day-to-day functions. This ‘synapse to network prodrome cascade’ posits that the major cause of non-motor dysfunction during prodromal Parkinson’s disease stems from the first scenario—synaptopathy driven aberrant neural network activity in the absence of overt cell death, which gives rise to clinical symptomology.

## α-syn is positioned to potently influence synaptic function

α-Syn is a 140 amino acid residue protein, encoded by the *SNCA* gene on Chromosome 4.^21^ α-Syn is ubiquitous in the brain,^22,23^ and cycles between a soluble unfolded monomeric state in the cytosol, and a membrane-bound α-helical multimeric state (dimer, trimer and/or tetramer).^24–29^ Binding to lipids and membranes stabilizes the helical multimers,^29^ rendering them resistant to aggregation.^30–34^ α-syn has two domains: (i) an N-terminal lipid binding domain (residues 1–95) and (ii) a highly acidic unstructured C-terminal domain (residues 96–140).^35–37^ The N-terminal domain consists of a hydrophobic non-amyloidogenic core that adopts an α-helical secondary structure to associate with lipid membranes,^24,35,36,38–41^ whereas, the C-terminal domain acts as a chaperone capable of interacting with other proteins and metal ions.^42^ Physiologically, α-syn serves to regulate the synaptic vesicle pool and trafficking, by aiding the formation of soluble N-ethylmaleimide-sensitive-factor attachment protein receptor (SNARE)-complex^42^ (for reviews see Burré^43^ and Sulzer and Edwards^44^). Vesicle fusion is mediated by SNARE proteins located on synaptic vesicles (i.e. synaptobrevin) interacting with the proteins on the presynaptic plasma membrane. Physiological α-syn directly interacts with synaptobrevin and phosphoinositols on the plasma membrane, chaperoning SNARE complex assembly.^42,45–47^ Apart from regulating vesicle dynamics *via* protein interactions, physiological α-syn is thought to play a role in the biosynthesis and homeostasis of monoamines,^48^ particularly dopamine,^49–55^ and in the regulation of synaptic glutamate release^56^ and synaptic plasticity.^57–59^

**Figure 1:**
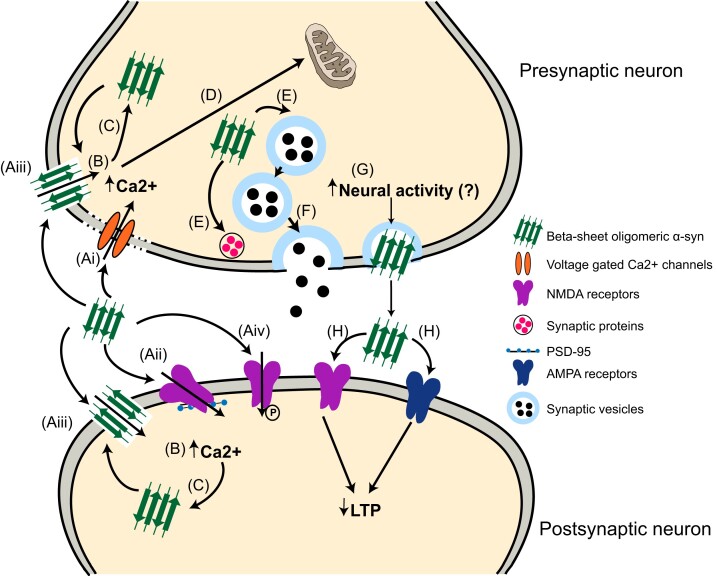
**Mechanisms of α-syn mediated synaptic dysfunction**. (**A**) Oligomeric α-syn interferes with the pre- and/or postsynaptic plasma membrane integrity by dislocating the (**Ai**) voltage-gated Ca^2+^ channels, (**Aii**) NMDA receptors, or (**Aiii**) by forming pore-like structures. Additionally, (**Aiv**) pathological α-syn phosphorylates and activates NMDA receptors. Together, (**B**) these pathological α-syn interactions lead to increased intracellular Ca^2+^ influx. (**C**) Increased intracellular Ca^2+^ levels can further stimulate α-syn aggregation and oligomer formation. Nevertheless, (**D**) due to the synergistic action of elevated Ca^2+^ and pathological α-syn, the mitochondria undergoes oxidative stress, potentially leading to cell death. (**E**) Presynaptic accumulation of pathological α-syn also interferes with the synaptic pool maintenance, and synaptic proteins, entailing (**F**) reductions in neurotransmitter release. Neuronal activity mediates the presynaptic accumulation and extracellular release of physiological α-syn, however, it is unknown if it applies to pathological α-syn. (**G**) Increased neuronal activity could result in an increase in the release of extracellular oligomeric α-syn. (**H**) Extracellular α-syn is further proposed to impair LTP *via* NMDA and AMPA receptors.

In contrast, under pathological conditions, the non-amyloidogenic core domain of endogenous α-helical, tetrameric α-syn destabilizes,^30^ and can adopt an insoluble β-sheet amyloid conformation to form pathological α-syn. This β-sheet conformation is associated with α-syn aggregation forming protofibrils, amyloid fibrils and ultimately Lewy bodies.^60–69^ During the early stages of Parkinson’s disease, pathological α-syn fibrils are predominantly formed in the neurites, eventually maturing into Lewy body like structures which are found in axon terminals.^70–72^ At the synapse, pathological α-syn interacts with synaptic machinery and disrupts synaptic signalling.^73^ This is followed by destruction of synapses, retraction of synaptic spines and the loss of neuronal connectivity, ultimately leading to cell death in later stages of Parkinson’s disease.^73–78^ This sequence of events associated with synaptic dysfunction which leads to neuronal death can take years.^79,80^ But, it is unknown how these events in the context of α-synucleinopathy ultimately result in the symptoms observed clinically. Nevertheless, the resulting pathological α-syn engages with specific presynaptic and postsynaptic components, which renders it a potent disrupter of synaptic function **(**Fig. 1). While the synaptic alterations in α-synucleinopathies have been thoroughly reviewed,^81^ we will discuss several key aspects and recent findings that are important to tie together, before establishing insight into the causes of network dysfunction.

## α-syn dysregulates neuronal Ca^2+^ dynamics

α-syn oligomers^82,83^ and elevated levels of intracellular α-syn^84^ are both known to promote Ca^2+^ transport into neurons. While high levels of intracellular Ca^2+^ promote the intracellular aggregation and oligomerization of α-syn,^85,86^ Ca^2+^ removal prevents intracellular aggregation.^85^ Thus pathological α-syn aggregation promotes Ca^2+^ dyshomeostasis, which in turn promotes further aggregation.

Models of α-synucleinopathy have yielded evidence for Ca^2+^ dysregulation.^82,84,87–94^ Pathological α-syn decreases plasma membrane lipid levels, leading to lipid-raft fragmentation. This fragmentation causes plasma membrane rigidity and affects the localization of ion channels and receptors integral to synaptic transmission.^95^ One such example of this involves N-,^92,96^ and L-type voltage-dependent Ca^2+^ channels,^97,98^ specifically Ca_v_ 2.2. Changes in lipid content force these channels to move into lower-lipid containing domains, and a shift from a closed to open state, thereby, resulting in increased presynaptic Ca^2+^-influx.^95,96^

Clearance of intracellular Ca^2+^ is achieved primarily by sequestering it either in the endoplasmic reticulum, or in the mitochondria; or directly pumping into the extracellular space *via* plasma membrane Ca^2+^ ATPase efflux pumps. Moreover, the mitochondria are responsible for ATP production required for Ca^2+^ efflux pumps. Pathological α-syn interacts with the endoplasmic reticulum and mitochondria compromising Ca^2+^ sequestration.^99–107^ Elevated intracellular Ca^2+^ levels further promote oligomeric α-syn aggregation, suggesting a vicious cycle between elevated cytosolic Ca^2+^ and α-syn aggregation.^107^

## Perturbation of cellular machinery, synaptic transmission and plasticity

Physiological α-syn is enriched in presynaptic nerve terminals,^23^ acting as a chaperone to maintain SNARE complex formation, which is responsible for controlling vesicle exocytosis.^42,108,109^ Recruitment of endogenous α-syn in the formation of pathological α-syn aggregates decreases its availability to engage in normal physiological functions.

Moreover, based upon work primarily in cultured hippocampal neurons, pathological α-syn alters the levels of several other synaptic proteins,^76,94,110,111^ and decreases levels of membrane lipids such as phosphoinositols,^45^ that regulate mechanisms of vesicle endocytosis and exocytosis. This suggests likely alterations in the number of vesicles. Physiological α-syn promotes normal clustering of vesicles at the synaptic terminal,^46^ thereby promoting SNARE complex formation. However, α-syn normally acts as a brake on the fusion of SNARE complexes and merging of synaptic vesicle membranes with the plasma membrane, thereby, preventing docking of the vesicles.^112^ Indeed, α-syn attenuates the mobility of synaptic vesicle pools between the presynaptic bouton and maintains the overall size of the clustered vesicles.^113^ In case of pathological α-syn inclusion formation, recruitment of α-syn away from the presynaptic terminal into aggregates releases this break and prevents vesicle clustering. This results in a decrease in the number of clustered vesicles,^109,111,114–117^ and conversely an increase in the number of docked vesicles.^93^ Alterations in neurotransmitter levels and/or the numbers of presynaptic vesicles released are reflected by changes in the frequency of miniature postsynaptic currents (mEPSCs). A recent report from Froula *et al*. demonstrated that pathological α-syn inclusions increase the frequency of mEPSCs, coupled with reduced Ca^2+^ transients in hippocampal neuronal cultures before any overt cell death. This evidence implicates an increase vesicle release probability as a likely factor contributing to altered synaptic transmission.^93^ On the contrary, others using similar cultures report a decrease in mEPSC frequency.^76,118^ This reduction is accompanied by an increase in the paired pulse ratio, which is reflective of increased presynaptic Ca^2+^ dynamics and therefore vesicle release probability. In this case, the dichotomous results could be primarily driven by increased presynaptic intracellular Ca^2+^ levels.^111^ Together, the effects on short-term plasticity are driven by pathological α-syn-induced disruptions in the presynaptic Ca^2+^ dynamics, and vesicle pool numbers and release probability. Alternative explanations for these changes in synaptic physiology could be compensatory mechanisms, based on evidence demonstrating alterations in: (i) the frequency of mEPSCs,^76,93,118^ (ii) numbers of dendritic spines^93,119,120^ or (iii) Ca^2+^ transient events.^93^ Regardless of the mechanism, these early disruptions of synaptic transmission are associated with presynaptic α-syn aggregates and perpetuates synaptic and postsynaptic dendritic loss as described below.^93,111^

While synaptopathy manifests in a cell intrinsic fashion, it can also act in cell extrinsic manners, including influencing postsynaptic neurons. Pathological α-syn can be released into the extracellular space in an activity-dependent fashion.^121^ This extracellular α-syn interacts with the postsynaptic neuron leading to a significant reduction in postsynaptic spine density and changes in morphology.^122^

Pathological α-syn interacts with postsynaptic neurons *via* several receptor mechanisms. Postsynaptic density modifications are associated with changes in N-methyl-d-aspartate (NMDA) receptor subunit composition.^123^ These changes in the composition can be attributed to either α-syn-induced impairment in synaptic transmission,^123^ or due to its direct interaction with the NMDA receptors.^124^ Pathological α-syn induces de-localization of postsynaptic membrane associated proteins, including PSD-95.^122^ PSD-95 is a scaffolding protein critical for membrane stability of NMDA receptors.^125^ Dislocation of PSD-95 induces movement of NMDA receptors, leading to altered receptor activity, and ultimately impairments in synaptic plasticity.^123,126^ Durante *et al*.,^127^ recently demonstrated that α-syn oligomers reduce the mEPSC amplitude in striatal spiny projection neurons—which indicates alterations in postsynaptic receptors. The impairments in long-term potentiation (LTP) are mediated *via* specific NMDA receptor subunits such as GluN2A^127^ and GluN2D.^128^ Misfolded α-syn also enhances α-amino-3-hydroxy-5-methylisoxazole-4-propionate (AMPA) receptor mediated synaptic transmission.^126^ In particular, impairment in LTP *via* inducing loss of GluA1-subunit bearing AMPA receptors in neurons overexpressing α-syn is observed.^129^ These impairments in LTP are indicative of decreased synaptic strength. Therefore, all these above studies support the view that synaptopathy is an early pathological consequence, before cell death in α-synucleinopathy.

It is important to emphasize that α-syn appears to act uniquely upon different neuronal compartments within individual neurons. Further, this impact appears to differ through the stages of Parkinson’s disease. In the prodromal phase, α-syn inhibits the mitochondrial Complex I in SN dopaminergic neurons.^99,130^ Loss of mitochondrial Complex I function is capable of driving axon loss, and *vice versa*. This forces the affected neurons to remodel their physiology by suppressing pacemaking, thereby narrowing action potential waveforms and downregulating Ca^2+^ entry and signalling.^130^ Similar circuit-level changes in α-syn-induced electrophysiological properties such as an increase in whole cell conductance and reductions in firing rate are reported.^131^ Further, localization of α-syn aggregates in the axonal processes,^132^ exacerbates this phenotypic downregulation and induces deficits in synaptic transmission, for example, by loss of synaptic proteins.^94,133,134^ These electrophysiological alterations can be partly remediated by blocking KATP channels (ATP sensitive K^+^ channels), suggesting that alterations in circuit function may occur before overt changes in synaptic transmission.^131,135^ Nevertheless, synaptic dysfunction subsequently expands retrogradely, affecting the somatodendritic compartment. These results suggest that pathophysiology in Parkinson’s disease is initiated within synaptic terminals and progresses proximally toward neuronal cell bodies in a ‘dying back’ fashion, thus reiterating that α-syn induces synaptopathy in the early stages of Parkinson’s disease. When damage crosses a threshold in the late stage of Parkinson’s disease, it leads to eventual death of neuronal perikarya. In the context of prodromal Parkinson’s disease, we propose the resultant early pathological onset and impaired synaptic transmission in brain regions and neurons burdened with pathological α-syn, leads to network dysfunction,^94^ often occurring concomitantly with the manifestation of non-motor symptoms^4,136–144^ as discussed below.

## Network dysfunction in Parkinson’s disease

**Figure 2:**
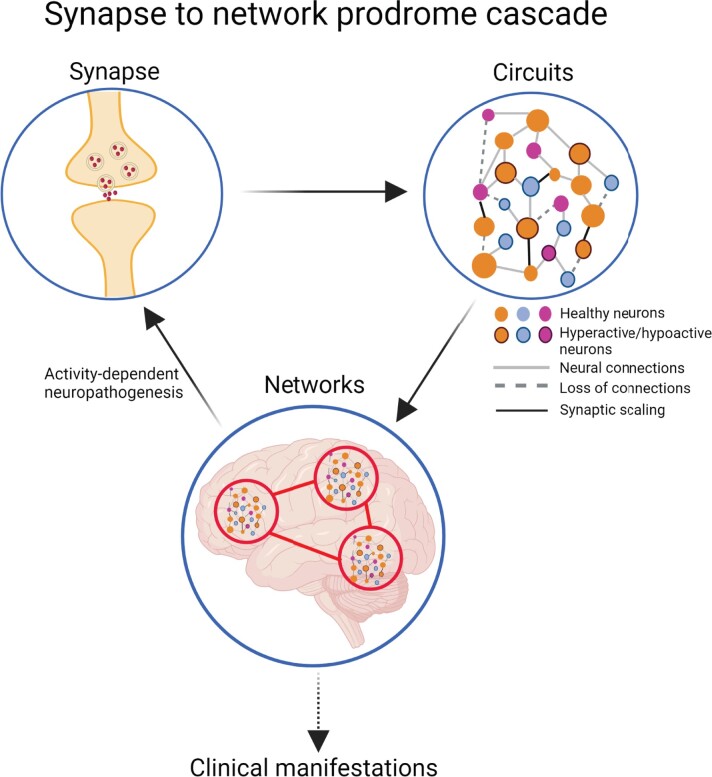
**Synapse to network prodrome cascade**. From the top-left: in the synapse, misfolded α-syn targets membranes, receptors, and crucial components of neural signalling pathways, driving synaptopathy (see Fig. 1). Loss of synapses affects synaptic communication, causing aberrant activity in individual neurons. This perturbed activity at the single neuron level, leads to the emergence of synaptic scaling mechanisms. Thus, impaired communication between neurons manifests as impaired communication within a circuit. Faulty circuit activity results in impairments at a network level. These aberrant network dynamics can then feedback to further influence synaptic function. In this model, these outcomes synergize to give rise to prodromal clinical manifestations including motor, sensory, and cognitive decline in α-synucleinopathies.

It is not the dysfunction of a single synapse, which is directly consequential for cognitive, sensory or motor function. Changes in multiple synapses ultimately lead to disrupted circuits and aberrations in large functional networks, which are considered the underlying drivers of major brain functions (Fig. 2). These distributed networks generate rhythmic oscillatory activity to communicate with one another, which can be measured within a brain region using local field potentials (LFPs), and at the skull surface through an EEG.^145^ LFPs are generated when neurons receive multiple synaptic inputs simultaneously, leading to synchronized oscillations.^146–150^ Thus, several forms of disruption such as desynchronization of oscillatory activity manifesting as either hyper- or hyposynchrony, both of which may impair communication within and between networks, and perturb function.^151–154^ Importantly, some evidence points toward alterations in the levels of synaptic proteins as underlying this desynchronization.^94^ Several lines of evidence link desynchronization of oscillatory activity to a variety of motor and non-motor symptoms discussed below.

Increases in beta-band network oscillations (∼15–35 Hz) in the basal ganglia and cortex are well documented in Parkinsonian primate and rodent models using LFPs,^155–158^ and Parkinson’s disease patients, commonly using electrocorticogram and electroencephalogram (for review see Brown^159^). While LFPs, electrocorticogram and EEG directly measure network-level electrical activity; functional MRI (fMRI) measures the indirect consequences of neural activity in the form of haemodynamic responses.^160^ fMRI has many strengths, including early disease detection, provides an understanding of prodromal and clinical symptoms, and predicts changes in clinical status as disease progresses.^161^ fMRI can also be used as a biomarker of alterations in brain physiology related to therapeutic interventions.^162,163^ This is important since fMRI can indicate changes in neural activity^164–166^ and connectivity,^167–170^ as well as regional differences in low frequency fMRI signals in the resting state in Parkinson’s disease patients.^165^ All of the above changes, whether monitored by LFPs, EEGs, electrocorticograms, or fMRI can importantly be linked to disease severity.^171^ Desynchronization manifesting as excessive synchronization is thought to contribute to pathological increase in beta-band activity, occurring along with motor deficits in monkey 1-methyl-4-phenyl-1,2,3,6-tetrahydropyridine models, as well as in parkinsonian individuals (for review see Refs.^172–176^). In line with this, normalizing beta synchronization using deep brain stimulation, or treatment with levodopa, improves motor function.^177–180^

Increases in beta-band activity are coupled with reductions in gamma-band oscillatory power in Parkinson’s disease, giving rise to exaggerated phase-amplitude coupling.^181–184^ Phase-amplitude coupling is a type of cross-frequency synchronization that represents information transmission and consolidation across multiple brain areas that are involved in pathologic and normal brain activities (e.g. Shimamoto *et al*.^185^ and Losacco *et al*.^186^). Exaggerated phase-amplitude coupling between beta and gamma oscillations is associated with Parkinsonian motor deficits.^187^

## Mechanisms underlying early network dysfunction: what we know and do not know

The above lines of synaptic and network-level insights together lead to the prediction that α-syn aggregation itself may influence neural network function. Emerging evidence from our lab and others links similar disruption in patterns of network oscillations to prodromal symptoms, using the early anatomical target—the olfactory system.^188,189^ An advantage of using the olfactory system is that the sensory information is monosynaptically conveyed from the OB into down-stream cortices. α-Syn aggregate formation in the OB results in olfactory perceptual dysfunction.^137,188,190–192^ Several lines of recent evidence have uncovered that injecting α-syn preformed fibrils in the OB,^189^ or virally expressing α-syn in the OB,^188^ both lead to aberrant local neural dynamics. This included changes in both beta- and gamma-band LFP activity as well as hyper-excitation of OB principal neurons. Recent work by Chen *et al*.^188^ suggests that α-syn aggregation in the OB neurons may be causal to these changes in neuron firing. Notably, in these models mice did not have cell loss.^188,190^ Consistent with these findings, additional recent work in a biophysical model of the OB during seeding-like damage, reported that decreasing the strength of synaptic connections in the OB leads to an increase in the power of oscillatory rhythms.^20,193^ Like the abnormal increase in beta-band activity observed in Parkinsonian individuals (described above), an increase in oscillations occurring as a consequence to α-syn aggregates could be detrimental to normal information processing of the OB and given the OB’s strong influence over down-stream connected structures (e.g. the amygdala, hippocampus) likely would impact other regions. While other early anatomical target structures including the brainstem, locus coeruleus and the raphe nucleus are suggested to contribute in late-stage Parkinson’s disease related motor impairments,^194–197^ their direct involvement in the generation of prodromal aberrant beta oscillations is not yet established.

The spatial component of the active neuronal ensembles, as well as the number and timing of action potential firing, together, encodes information subserving behaviour.^198–200^ Even individual synapses appear important for promoting synchrony depending on the firing frequency of the neurons,^201–203^ intrinsic properties of the neurons^203–205^ and location of these synapses on the dendritic trees.^205,206^ Therefore, any alteration in the patterns of neuronal firing, as observed in the subthalamic neurons in both Parkinsonian monkeys and persons with Parkinson’s disease (e.g. Refs.^207–209^) can dramatically shape network activity. This becomes a complex problem in the context of prodromal α-synucleinopathy, especially since we do not yet fully understand which neuron populations accumulate pathological α-syn. Beyond that, local neurons, both inhibitory and excitatory, may form a variety of circuitry among each other (recurrent, feedforward, feedback). Many brain circuits are also subject to centrifugal and/or neuromodulators input from other brain areas (locus coeruleus, raphe nucleus, substania nigra, etc.). While not completely elucidated, computational studies suggest that an increase in inhibitory interneuron spiking and the resultant decrease in the firing of the glutamatergic projection neurons, drives generation of beta oscillations.^210,211^ That stated, the exact mechanism(s) underlying the emergence of pathological beta oscillations are unclear, but include several candidates (Table 1).

**Table 1:** Candidate mechanisms linking elevated beta-band activity with excitation-inhibition imbalance

*Intrinsic alterations* Inhibitory interneurons maybe more susceptible to: (i) α-syn accumulation,^212^ and/or (ii) higher excitability due to increased synaptic conductance, i.e. elevated Ca^2+^ influx *via* NMDA and Ca^2^ + channels.^211^ Larger numbers of interneurons participating in the beta oscillations. Increased oscillatory coherence between interneurons. *Top-down alterations* α-Syn accumulation in neighbouring brain regions resulting in compensatory input to interneurons, increasing their spiking. *Bottom-up alterations* α-Syn increases conductance of projection neurons, sufficiently exciting the interneurons such that the latter induces a strong inhibitory input silencing the former. Combinations of the above intrinsic, top-down, and bottom-up alterations.

Based on the above discussed evidence, we know that α-syn-induced synaptic and network-level perturbations begin relatively soon after disease onset, however, the prodromal symptoms can extend for more than two decades before tremors and massive cell death emerges. So, what cellular or network mechanisms could account for this prolonged prodromal stage? One hypothesis, as outlined below, is for a role of synaptic scaling.

## A consideration for synaptic scaling

In late stages of Parkinson’s disease, one model is that certain vulnerable neurons die, leading to either hyper- or hypo-excitability among interconnected neurons, thereby resulting in network-level changes. Yet in the early stages of Parkinson’s disease, rather than inducing cell death, pathological α-syn induces changes in plasticity through several mechanisms including interfering with NMDA receptor subunit composition,^123,126^ NMDA receptor trafficking,^213–216^ changes in spine density^217^ and targeting vital mitochondrial components,^218^ to name a few. These changes in plasticity certainly may entail altered neuronal excitability and synaptic strength. Additionally, the late-stage Parkinson’s disease conceptual model is seated largely in short-term changes of activity (for example in Parkinson’s disease, decrease in dopamine levels decrease D2 receptor signalling, thereby, increasing the excitability of indirect pathway neurons leading to hypokinetic movement). As such, the late-stage model does not fully account for the natural tendency of neurons to establish homeostatic plasticity as demonstrated in some recent Parkinson’s disease models.^120,219^ When neural activity is perturbed for a sustained duration, it induces homeostatic adaptations in the synapse that restores activity of a given neuron to a set point.^220,221^ Homeostatic plasticity is achieved by either of two mechanisms. First, changing the intrinsic excitability: neurons express a different constellation of ion channels that shifts their ability to fire in one direction or the other (more excitable versus less).^222^ Or two, scaling synaptic strength: for example, in case of a lower firing rate, synaptic strength becomes greater and the cells will begin to fire at a homeostatic rate. Conversely, if one raises firing rates, synaptic strength will decline.^223^ In the early stages of Parkinson’s disease, before significant neuronal death, it is possible that the changes in synaptic strength drive circuits to establish homeostatic plasticity, ultimately precipitating at a network level.

Regulating the firing of individual neurons *via* network rearrangements is another strategy to maintain circuit homeostasis. Cognitive defects observed in the prodromal stages of α-synucleinopathy,^224,225^ are associated with impaired brain functional connectivity.^226–232^ Some studies report a decrease in functional connectivity,^233,234^ whereas others report an increase.^235^ Generation of aberrant oscillations could be a result of a compensatory increase in functional connectivity. Another possibility for the generation of aberrant oscillations is that the homeostatic adaptations fail and that the functional damage to the neurons is too great to compensate. Therefore, these homeostatic forms of synaptic plasticity could be important determinates for the network-level perturbations in the early stages of α-synucleinopathies. The idea of compensatory mechanisms at play in prodromal Parkinson’s disease was recently supported by a study using human neural networks.^236^ Therefore, we speculate that the increase in the pathological oscillations may result from compensatory mechanisms in the early stages of the disease, which consequently delay the manifestation of clinical motor symptoms. Since loss of 50–60% of neurons is required for motor symptoms to emerge,^237^ synaptic scaling could increase the neurodegenerative threshold in prodromal Parkinson’s disease. Nevertheless, once neurodegeneration reaches a particular level of severity, it cannot be counteracted by compensatory mechanisms and functional deficits ensue.^238^

## Specificity within circuits resulting in network dysfunction

Although a wide variety of neurons can accumulate pathological α-syn,^239–245^ not all neurons show pathological accumulation. Based upon the Braak hypothesis, all the neurons that are anatomically connected to the neurons with pathology should be vulnerable. However, there is considerable variation in the temporal pattern of α-syn accumulation and severity of neuronal damage than originally hypothesized by Braak. Several cell-autonomous factors have been proposed which could help identify at-risk neurons. Thus, it is suggested that the spreading pattern of α-syn-induced pathology and dysfunction occurs as proposed by Braak, but further that spreading to a specific population of neurons is dictated by a cellular-phenotype that renders them less able to cope with the burden of pathological α-syn.

These cell-autonomous factors can be broadly divided into two categories: neuroanatomical and/or molecular factors, and physiological factors. Molecular factors include the amount of molecular templates including endogenous α-syn available to misfold. For example, glutamatergic neurons expressing higher levels of endogenous α-syn are especially vulnerable to α-syn accumulation.^246–250^ Further, glutamatergic neurons express lower levels of BAG_3_, which is involved in autophagy.^251^ Lower BAG_3_ expression may lead to reduced α-syn degradation, in turn leading to increased accumulation.^252^ Additionally, in the α-syn seeding model of prodromal Parkinson’s disease, glutamatergic neurons exhibit Math_2_^+^ loss, a transcription factor underlying regulating mitochondrial homeostasis. This further increases vulnerability of these neurons to α-syn.^250^ Additionally, GABAergic neurons expressing parvalbumin and calbindin, playing a role in Ca^2+^ buffering and signalling, are also less vulnerable to inclusion development.^253–255^ Together, accumulation of aggregates in glutamatergic and GABAergic neurons might interfere with their physiological activity,^93,127,256^ leading to network-level changes. Neuroanatomical factors include hyperbranching of neurons with a large number of release sites. Indeed, hyperbranching of long projection axons is observed in almost all α-syn aggregation-prone neurons, regardless of transmitter identity.^257,258^ Hyperbranching of long projection axons is observed among SN dopamine neurons, dorsal motor nucleus cholinergic neurons, locus coeruleus noradrenergic neurons and raphe nucleus serotonergic neurons.^259–263^ Mitochondrial stress, a major driver for dysfunction and eventual neurodegeneration, is elevated in the axons of these vulnerable neurons^264^ due to the large axonal network that requires metabolic support and the high energy demand caused by neurotransmitter release.^262^ However, not all neurons that have highly branched axons are vulnerable.^265^ Thus, this trait alone is not sufficient to induce vulnerability.

The second factor that is an important determinant of vulnerability is the physiological phenotype of the neurons. At-risk neurons are particularly slow autonomous pacemakers—having low frequencies of firing (∼2–5 Hz) with relatively broad action potentials (∼4 ms width). Most neurons rely on Na^+^ permeable channels to drive pacemaking. However, vulnerable SN dopaminergic neurons rely heavily on L-type voltage-gated calcium (Ca^2+^) channels, allowing large Ca^2+^ influx into the cytoplasm.^266^ Due to pathological α-syn mediated enhancement in L-type Ca^2+^ channel activities,^95^ there is a higher increase in Ca^2+^ influx, rendering the dopaminergic network vulnerable.^107^ Additionally, these neurons express low levels of Ca^2+^ buffering capacity.^267^ The sustained Ca^2+^ influx and Ca^2+^ oscillations create a bioenergetic burden, increasing oxidative stress in the mitochondria. The mitochondria, thereby, produce reactive oxygen species,^268^ detrimental for neuron health.^102^ These physiological features such as prominent transmembrane Ca^2+^ currents, broad action potentials and low intrinsic Ca^2+^ buffering capacity are shared by cholinergic neurons of the dorsal motor nucleus, noradrenergic neurons of the locus coeruleus, dopaminergic neurons in the OB and the serotonergic neurons of the raphe nucleus impacted early on in the disease.^91,269–276^ Taken together, depending on the neurons that are affected, where they project to, and how their responses are gated, ultimately impacts how they modulate large-scale network dynamics that they are a part of.

## Beyond prodromal: the path to cell death

Axonal degeneration and loss of synaptic connections occur in parallel to cognitive impairments.^277–279^ Conversely, restoring synaptic dysfunction normalizes aberrant brain activity associated with cognitive decline.^280–284^ Together, this suggests that synaptopathy precedes neuronal loss *in vivo*,^285^ and in individuals with Parkinson’s disease.^286–290^ This additionally suggests that at least in the context of cognitive deficits, it is the subtle synaptic changes rather than overt cell death which underlies the network-level perturbations driven by α-syn aggregation.

How does cell death fit in the sequence of events leading to the functional deficits during the Parkinson’s disease prodrome? We propose a pathological cascade wherein synaptopathy leads to aberrant network activity, occurring concomitantly with the non-motor prodromal deficits in the absence of cell death.^291^ As the disease progresses over the course of several years, sustained and large-scale aberrant neural dynamics drive vulnerable neurons past their ability to maintain homeostatic plasticity and over the neurodegenerative threshold, exacerbating the preexisting impaired network dynamics, ultimately leading to motor deficits.

Specific lines of evidence support this model leading to cell death. Axonal degeneration due to accumulation of misfolded α-syn^94^ eventually promotes neuronal death.^292,293^ For example, α-syn oligomers can prolong NMDA receptor activation and increase intracellular Ca^2+^. Influx in Ca^2+^ leads to calpain activation, promoting excitotoxicity^294,295^ and disruptions in connectivity.^94^ Augmented synaptic transmission *via* AMPA receptors also contributes to excitotoxicity and cell death.^296^ Because pathological α-syn can impair mitochondrial function^297,298^ and induce oxidative stress,^299,300^ Ca^2+^ buffering becomes difficult, leading to cell death.^253^ Moreover, alterations in synaptic function can also initiate apoptotic pathways.^79^ Therefore, synaptic alterations manifesting as circuit and network-level perturbations may underlie cell death.

Overexpression of α-syn *in vivo* requires a long period of time and high level of expression to induce neuronal death.^285^ Recently, using a α-synucleinopathy model of prodromal Parkinson’s disease, Mahul-Miller *et al*. proposed that Lewy body formation, and not fibril formation, is one of the major drivers of neurodegeneration.^70,72^ Therefore, in prodromal Parkinson’s disease, it is possible that α-syn aggregates cause synaptic and neural activity changes, but not neurodegeneration and cell death.

Together, since pathological burden and neuronal loss are not always positively correlated,^301^ the subtle effects of pathological α-syn on synaptic function could explain cell death. Further, axonal degeneration co-occurring with prodromal deficits precedes neurodegeneration.^277–279^ This suggests that at least in the context of early α-synucleinopathy, loss of synapses, as opposed to than death of neurons, correlate much more strongly to functional deficits.

## α-Synucleinopathy and neural dysfunction: future perspectives and challenges

Multiple factors can contribute to impaired neural dynamics, however, there are some gaps in the literature (Table 2**)** that if answered could enhance our knowledge about prodromal Parkinson’s disease-associated dysfunction.

**Table 2:** Outstanding questions to understand synaptic and network causes underlying the Parkinson’s disease prodrome

What is the role of glial pathology and how does it affect neural activity? What is the relationship between pathological burden, network dysfunction, and clinical manifestations? What is the role of other cellular dysfunctions (e.g. neuroinflammation, oxidative stress, mitochondrial dysfunction) on the neuropathogenesis of α-synucleinopathies? What specific cell types are functionally vulnerable to and/or aggregate α-syn pathology? Why are they vulnerable? How does activity-dependent neuropathogenesis contribute to perturbed network dynamics? Does deep brain stimulation entail long-term changes to neural networks?

While we have some understanding of the pathogenic roles of α-syn in neurons (as discussed above), we know very little about the functional impacts of α-syn assemblies on the immune cells. Further, their contribution to the uptake and spreading of α-syn is poorly understood. While microglia and astrocytes can contribute to phagocytic clearance of pathological α-syn,^302,303^ glia also appear involved in the transmission of pathology,^304^ in part *via* glial exosomes^305^ and possibly *via* tunnelling nanotubes.^306^ α-Syn fibrils can trigger activation of microglia, that might contribute, together with astrocytes,^307^ to neurotoxicity and release exosomes carrying pathological α-syn.^305^ Further, microglia activation can lead to dysregulated neuronal Ca^2+^ responses and increases the probability of neuronal death by excitotoxicity.^308,309^ Therefore, insights into how these non-neuronal cells are affected by α-syn is key to elucidate their role in the large-scale impaired network dynamics.

There is also a need to establish a relationship between pathological burden, network dysfunction and clinical manifestations. This has been particularly challenging due to the lack of reliable biomarkers of disease progression, however, recent advances are aiming to fill this void.^310^ Clearly, other mechanisms in Parkinson’s disease beyond α-syn and death of neurons and occurring either in parallel or independently, could influence the network dynamics leading to the development of clinical manifestations in prodromal α-synucleinopathies. Some of these include neuroinflammation, oxidative stress and mitochondrial respiration defect, which all can trigger neuronal dysfunction (for reviews see Refs.^311–315^). Most likely, cell-autonomous factors including the degree of arborization, bioenergetic requirements and their intrinsic milieu make different neuron types more or less vulnerable.^100^ Whether certain neurons are affected late or early in the disease process are also influenced by their connectivity, and how they are connected to the areas that are impacted first by α-syn aggregates. Trans-synaptic spread includes retrograde and anterograde transmission, and both might contribute to the pathological progression to different degrees.^246,316^ Further, determining if these cells are hyperactive or hypoactive will help elucidate their specific contributions to the impaired brain dynamics.

The association between neuronal activity and the spread of α-syn pathology is poorly understood. Physiological, natively unfolded α-syn is released by primary neurons^317^ and extracellular α-syn in the brain interstitial fluid seems to depend on neural activity.^318^ Recently, Wu *et al*.^121^ reported the spread of pathological α-syn due to synthetically enhanced neuronal activity. However, the contribution of activity-driven release of aggregation-prone α-syn species that can propagate to neighbouring neurons is unknown. This question is especially relevant due to the current use of deep brain stimulation to correct perturbed pathological network oscillations. Stimulation of neural network activity does not necessarily restore network activity to pre-pathological states but, rather to a third state that alleviates functional improvement relative to diseased state, but that might not necessarily be normal.^319^ Additionally, restorative action could increase connectivity.^320^ Together, one can still ask what effects does enhancing neural activity *via* deep brain stimulation have on the spread of pathological α-syn in networks with enhanced connectivity.

Finally, many parallels can be drawn between Parkinson’s disease and other protein aggregating neurodegenerative diseases such as Alzheimer’s disease. Alzheimer’s disease involves two major kinds of insoluble protein aggregates including extracellular aggregates comprised of amyloid-β, which shares the non-amyloidogenic core with α-syn, and intracellular aggregates of tau.^321^ Pathological Aβ (for review see Palop and Mucke^322^ and Wesson *et al*.^323^) and tau^324^ disrupts synaptic transmission strength and/or long- and short-term plasticity, preceding neurodegeneration. The underlying mechanisms may involve alterations in Ca^2+^ homeostasis, endoplasmic reticulum stress, increased levels of reactive oxygen species and activate kinases, caspases and calpains (for review see Small *et al*.^325^). Therefore, the loss of functional synapses entails profound effects on Alzheimer’s disease symptomology.^325–327^ Further, at the circuit and network levels, these pathological aggregates can elicit early aberrant increases in neural activity,^328^ which has been demonstrated in cell culture, cortical brain slices, and *in vivo*,^329–334^ suggesting both direct proexcitatory effects on glutamatergic neurons and impairments of inhibitory interneurons.^322,329,335^ Overexcitation of neural networks triggers compensatory responses including extensive remodelling of circuits,^329^ and synaptic scaling mechanisms to alter synaptic function (for review see Small *et al*.^336^). Therefore, these protein aggregating diseases appear to share a common overarching theme which involves synaptic-, circuit-, and therefore network-level impairments underlying symptomology. Further studies on the progression of these mechanisms during preclinical stages could reveal new targets for therapeutic drug development.

## Conclusions

Taken together, in the stages of α-synucleinopathy that are linked to prodromal Parkinson’s disease, misfolded α-syn impacts neural function, ultimately leading to perturbed network-level activity. It is reasonable therefore, although it has not been established that this precipitates into impaired function and the emergence of debilitating non-motor symptoms—the underlying premise of the ‘synapse to network prodrome cascade’. However, it is important to emphasize that the direct relationship between network dysfunction and clinical manifestations is far from clear. Gaps in our understanding of α-syn pathogenesis are obstacles to the development of therapeutic approaches that target symptoms in the Parkinson’s disease prodrome. Information processing across distributed networks is critical for our daily functions, and understanding the mechanisms underlying impaired network dynamics will have a large impact in our conceptualization of Parkinson’s disease. The major advantage of studying the underpinnings of these functional impairments early in the disease progression cascade is the possibility to prevent irreversible cell death, especially among dopaminergic neurons which are paramount, especially to non-prodromal Parkinson’s disease. Altogether, systems neuroscience approaches when combined with a range of *in vivo* models—from small animal to human—will pave the way for great advances in understanding and treating Parkinson’s disease and other α-synucleinopathies.

## Data Availability

Data sharing is not applicable to this article since no new data were generated nor were any data analyzed in this study.

## Abbreviations

α-syn =: α-synuclein
Ca^2+^ =: calcium
LFP =: local field potential
LTP =: long-term potentiation
mEPSC =: miniature postsynaptic current
OB =: olfactory bulb
SN =: substantia nigra
